# Time to Blindness and Associated Factors Among Glaucoma Patients Under Treatment at Governmental Hospitals in Amhara Region, Ethiopia; Application of Gamma Shared Frailty Accelerate Failure Time Model

**DOI:** 10.1002/hsr2.72016

**Published:** 2026-03-08

**Authors:** Awoke Seyoum Tegegne, Nurye Seid Muhie

**Affiliations:** ^1^ Department of Statistics Bahir Dar University Bahir Dar Ethiopia; ^2^ Lecturer in Bio‐Statistics, Department of Statistics Mekdela Amba University Tulu Awulia Ethiopia

**Keywords:** AFT, gamma shared frailty, glaucoma, intraocular pressure, time to blindness

## Abstract

**Background and Aims:**

In Ethiopia, glaucoma and related cases account for 5.2% of the irreversible blindness and is the fifth main cause of blindness. The aim of this study was to investigate the time to blindness and associated factors among glaucoma patients under treatment at government hospitals in Amhara region, Ethiopia.

**Methods:**

A retrospective cohort study using a gamma shared frailty accelerated failure time model was conducted at 5 randomly selected public hospitals in Amhara region. About 1102 glaucoma patients, whose follow ups, from January 2018 to December 2023 were randomly selected using stratified random sampling technique.

**Results:**

The results of the multivariable Gamma Shared Frailty accelerate failure time model indicates that age [ϕ = 0.694; 95% CI: (0.408, 0.773)], literate glaucoma patients [ϕ = 1.163, 95% CI: (1.062, 1.206)], urban glaucoma patients [ϕ = 1.157; 95% CI: (1.049, 1.274)], non‐hypertensive glaucoma [ϕ = 0.812; 95%: (0.673, 0.979)], non‐diabetic glaucoma patients [ϕ = 0.949; 95% CI: (0.533, 0.687)], housewives [AOR = 0.772; 95% CI: (0.428, 0.975)], farmers Glaucoma patients (ϕ = 0.969; 95% CI: (0.735, 0.999)], moderate glaucoma stage [ϕ = 0.791; 95% CI: (0.419, 0.890)], advanced stage glaucoma patients [ϕ = 0.959; 95% CI: (0.703, 0.998)], glaucoma patients with cup‐disk ratio (CDR) > 0.7 [ϕ = 0.931; 95% CI: (0.739, 0. 998)] and glaucoma patients with normal IOP (ϕ = 1.058; 95% CI: (1.019, 1.285)] significantly affected the time to blindness for glaucoma patients.

**Conclusion:**

Age, lack of education, and residing in a rural area were all associated with a shorter time to blindness. Conversely, patients with better IOP control, no concurrent hypertension or diabetes, and those with a lower cup‐to‐disc ratio (CDR) showed a longer time to blindness. The significant frailty effect confirmed that there is substantial variation in patient outcomes across different hospitals, likely due to differences in healthcare quality and provision. To minimize this disparity and ensure consistent, high‐quality care, it is critically important to standardize treatment protocols across all facilities.

**Abbreviations**
AFT: accelerated failure time
AIC: akaike information criteria
BIC: Bayesian information criterion
PHs: proportional hazards
WHO: World Health Organization.

## 1 Background

Globally, glaucoma accounts for the second most common cause of blindness and especially, open‐angle glaucoma was a cause of irreversible blindness for about 5.9 million people by 2020 [1]. It is a significant public health issue due to the substantial increase in the projected number of glaucoma cases within the next several decades [2].

Glaucoma is a significant portion of blindness and public health problem in Africa, with a high prevalence and a substantial impact on blindness, especially for sub‐Saharan region [3]. It is responsible for 15% of blindness cases in Africa [4]. The problem of glaucoma is acute and major health issue in sub‐Saharan Africa (SSA) [5]. Glaucoma in Africa occurs at an earlier age and progress more rapidly compared to other regions [2]. As, Sub‐Saharan Africa progresses through the demographic transition from low mean life expectancy to higher life and form high birth rates to lower birth rates, age‐related eye diseases such as glaucoma, rapidly increase in the region [6].

In Ethiopia, where 85% of the population is in rural areas whose major activities is agriculture, and glaucoma is considered as leading cause of blindness, contributing to a significant portion of irreversible vision loss [7]. According to the Ethiopian National Blindness and Low Vision Survey, it is known that glaucoma is responsible for about 62,000 individuals in Ethiopia [8]. This result further indicates that there is a concerning situation with limited access to early detection for corrective measures related to treatment, low awareness and limited ophthalmological services in the country.

In the Amhara region of Ethiopia, glaucoma is a significant cause of blindness, with studies showing, it contributes to a substantial portion of overall blindness, and awareness and access to care remain challenges [9]. Majority of the population are engaged in agriculture where dust particles entered to the eyes of an individual and suffer from visual related diseases such that glaucoma, Trachoma and the like [7]. As the leading cause of irreversible blindness in the world, glaucoma poses a significant public health problem in the study area, Amhara region [10]. Lack of awareness about glaucoma is an important reason for its late demonstration which significantly increases the risk of blindness [11]. Awareness about its nature and risk factors is known to affect the behavior for seeking intervention [12]. Raising public awareness and knowledge of glaucoma is a key means of addressing the overwhelming consequences of the disease [13].

Despite the fact that, a large amount of time and resources spent on treatment of glaucoma, there are still few effective treatments used to reduce the incidence of glaucoma and related issues [14]. Even though there are some health professionals in the rural areas of the region, awareness and understanding of glaucoma is low among the community [15]. In the region, many instances of parents defend that their child does not have an eye problem while in fact they are suffering from congenital glaucoma and many people are suffering with acute angle‐closure glaucoma who have been treated for conjunctivitis [16]. The burden of blindness from glaucoma, specifically primary open‐angle glaucoma (POAG), highly affects the visual status of peasants in the rural areas in the study area (Amhara region of Ethiopia) [17].

Due to the nature of the disease, an inadequate and inaccessible health institutional service, and a very poor level of public awareness, glaucoma patients tend to come for help after they have become either unilaterally or bilaterally blind [18]. In general, evidence‐based information is lacking regarding the proportion and contributing factors of glaucoma in Ethiopia particularly, in the study area, despite the existence of the problem related to the variable of interest [19].

Multiple studies have been conducted, but these findings are based on studies conducted outside Africa with studies yielded lower prevalence rate of glaucoma [20]. Possibly, because of the genetic heterogeneity of groups of people of African descent and the fact that most African Americans and Afro‐Caribbeans are descendants of West and not East or Southern, the study results obtained inside Africa may be different from studies conducted outside African regions [21]. In Africa, with the considerable genetic heterogeneity in SSA people, it is less likely that existing studies in East and South Africa are the same to all Sub Saharan African populations [22].

Few studies have been also conducted previously about the variable of interest in Ethiopia. However, such studies are restricted in a single health institution such as hospital (Small catchment areas) and not as regional based studies including many health institutions or hospitals [23]. Including large catchment area like region wide research may help to develop regional policy implications. Hence, the region wide study may give integrated policy implication as the country categorized by regions with widest area as compared to zones or institutions. Most of the researches conducted previously considered are a simple survival model without considering the shared frailty term. Such studies have highlighted the need for further studies on the time to blindness and associated factors, considering region wide studies and potential predictors not included in their studies [24].

The above gaps became a motivation for the current investigation. To overcome, these gaps, in the current study, the time to blindness among glaucoma patients under treatment were clustered by the hospitals in the region with its catchment area for time to blindness by introducing the shared frailty term in the usual survival model considering hospitals or zones within the regions as clusters. The shared frailty term was added to account the correlation that existed among the clusters of latent random effect.

The current study used a parametric shared frailty accelerated failure time (AFT) models like Weibull, exponential, log‐logistic, and log‐normal distributions to compare and get the best model which fits the time to blindness and associated factors. Therefore, the aim of this study was to investigate the time to blindness and associated factors among glaucoma patients under treatment at government hospitals in Amhara region, Ethiopia.

The purpose of this study is to establish the average time to blindness for glaucoma patients in the Amhara region. This is a critical metric because it provides a realistic timeline for disease progression within this specific patient population. By knowing this, healthcare providers can better counsel patients and set realistic treatment expectations. The study's most crucial objective is to pinpoint the factors that accelerate or delay the progression to blindness. For the effective policy formulation and motivation of glaucoma patients to alive for a longer period of time with their normal eye visual, it is crucial to study the various Socio–economic and demographic factors affecting the time to blindness. The findings can directly inform public health strategies. This study is also important for the Ministry of Health and the local Regional Health Bureau to draft a policy on the prevention and control of complications associated with glaucoma. This study can also serve as an audit of the current healthcare system's effectiveness. It can reveal potential gaps in care, such as long waiting times, lack of specialized equipment, or inadequate follow‐up protocols, which may be contributing to vision loss despite patients being “under treatment.”

## 2 Materials and Methods

### 2.1 Study Area

**Figure 1:**
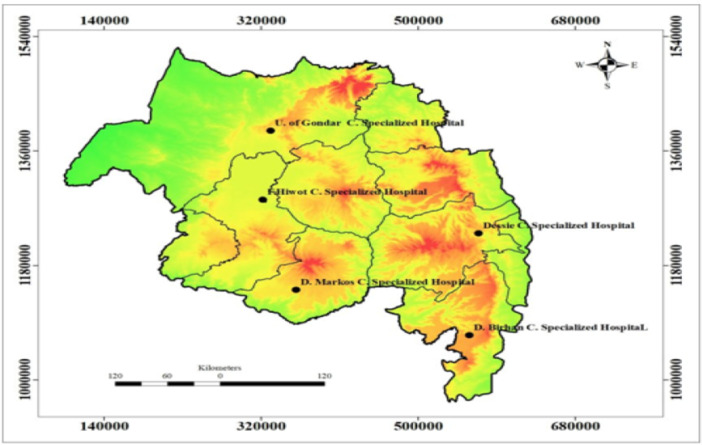
Location of five governmental hospitals in Amhara region.

The study was conducted in 5 governmental hospitals in Amhara region namely Felege‐Hiwot Comprehensive Specialized Hospital, University of Gondar Comprehensive Specialized Hospital, Dessie Comprehensive Specialized Hospital, Debre Birhan Comprehensive Specialized Hospital and Debre Markos Comprehensive Specialized Hospital (Figure 1).

### 2.2 Study Design and Period

An institutional‐based retrospective cohort study design from 2018 to 2023 was conducted for the current study. The participants under this study were glaucoma patients who enrolled in the first ten months of 2018 and followed up to 2023.

### 2.3 Study Population

The study population included under the current study were all glaucoma patients registered at the Ophthalmology clinic at each of the 5 hospitals in Amhara region, north‐west Ethiopia whose follow ups were 2018–2023.

### 2.4 Inclusion and Exclusion Criterion

Each patient was followed retrospectively from the start of glaucoma treatment until end of study time. Hence patients with full information about their follow ups with clinical outcome variables and socio‐demographic variables were included under the current study. Those patients who stopped the prescription medication for any reason during treatment, patients who did not have full recorded information regarding the status of glaucoma were excluded.

### 2.5 Sample Size Determination Procedures

About 5 governmental hospitals were considered for this study. These hospitals were selected using purposive sampling techniques, considering high number of glaucoma patients under treatment in the region. In these hospitals, about 6500 glaucoma patients were under treatment. Among these patients, patients having measures of status of glaucoma, having full follow ups (full records) and those with full inclusion criterion were considered for data analysis. Hence, a total of 1102 study participants were selected using stratified random sampling technique from each hospital. The total number of glaucoma patients and samples in each hospital are indicated in Table 1.

**Table 1:** Samples from each of the five hospitals.

Name of hospital	Total glaucoma patients	Sample size
Felege Hiwot Comprehensive Specialized Hospital	1745	296
University of Gondar Comprehensive Specialized Hospital	1250	212
Dessie Comprehensive Specialized Hospital	890	151
Debre Birhan Comprehensive Specialized Hospital	1345	228
Debre Markos Comprehensive Specialized Hospital	1270	215
Total	6500	1102

### 2.6 Data Collection Procedure

The clinical outcomes during data collection were taken from both left and right eyes and socio‐demographic and clinical information were collected by the health staff at treatment time. For the glaucoma patients under study, the important variables and corresponding data were extracted in the charts/cards of each patient. Hence, the important variables and their values were collected in each patient's chart and electronic database system was applied in data collection, considering id number of each patient in each of the five public hospitals. The data were retrieved by trained ophthalmologist data clerks based on inclusion criteria.

### 2.7 Data Quality

To get high standard quality data, 2 day training was given for data collectors. Data collection was conducted by well‐trained data extractors. To get quality data in data collection process, the principal investigator had close follow ups and checked the checklists for completeness and consistency of daily activities.

### 2.8 Response Variable

The response variable for the current study was time to blindness for glaucoma patients under treatment measured in months.

The time to event was considered from the time of commencement of treatment to study period. In this study, blindness is almost always defined by a threshold of legal blindness or functional blindness (visual acuity of less than 20/400 or a severely restricted visual field in the better‐seeing eye). The outcome time to blindness in glaucoma survival analyzes is most commonly defined based on the better‐seeing eye as an event for the patient. This measures the patient's overall functional vision. A patient is considered blind when the visual acuity in their better eye meets the threshold for legal or functional blindness. The event (blindness) occurs when the patient reaches this threshold in their better‐seeing eye [25].

### 2.9 Independent Variables

The predictor variables considered under this study were sex (male, female), age in years, residence areas (rural, urban), marital status (living with partner, living without partner), level of education (literate, illiterate), job type (farming, government work, home wife, private business), existence of Hypertension (yes, no), type of treatment (Timolol, Timolol and Pilocarpine, Timolol and Diamox, Timolol, Pilocarpine, and Diamox), stage of glaucoma (early, moderate, advanced), duration of treatment (short, medium, long), Cup‐disk ratio (0.7, > 0.7) and existence of diabetes (yes, no).

### 2.10 Methods of Data Analysis

#### 2.10.1 Semi‐Parametric Models

The Cox‐Proportional Hazard Model was used for estimating time‐varying, time‐independent, continuous, and discrete covariates. Kaplan‐Meier estimate and log‐rank test were also used for all categorical variables to know whether there is a significant difference among the categories of each predictor variable. Cox proportional hazard model was also fitted to examine the effects of different predictive factors on the time to blindness of glaucoma patients [26].

#### 2.10.2 Survival Function

The survival function S(t) is defined as the probability that the survival time of a randomly selected subject is greater than or equal to a specified time, t [27]. Thus, it gives the probability that an individual survives beyond a specified time.

Let T be a random variable associated with the survival times, let t be the specified value of the random variable, and let f (t) be the underlying probability density function of the survival time T. The survival function, S (t) is given as [28].

S(t)=p(>t=1−F(t)),t≥0,

where F (t) is the cumulative distribution function, which represents the probability that a subject selected at random would have a survival time less than or equal to some sated value t.

$$F(t)=p(T\leq t)=\int_{0}^{t}f(u)du,\;t\geq 0.$$

The relationship between S(t) and F(t) is given as

$$f(t)=\frac{d}{dt}F(t)=\frac{d}{dt}(1-S(t))=-\frac{d}{dt}S(t),\;t\geq 0.$$

#### 2.10.3 Hazard Function

The hazard function is widely used to express the risk or hazard of blindness at some time t and is obtained from the probability that an individual with blindness at time t, conditional on he/she having survived to that time. The hazard function, typically denoted as h(t), represents the risk or likelihood of a patients having blindness at a specific time t [29]. It quantifies the instantaneous rate of the event per unit of time, assuming that the individual has survived up to time t. The hazard function is derived from the probability that an individual experiences blindness within a small interval (t, ∆t), given that they have survived up to time t [30].

The hazard function considers the probability that the random variable associated with an individual's survival time, T lies between t and t +∆t, conditional on T being greater than or equal to t, written as P (t ≤ T < t + ∆t/T ≥ t) [31]. It is the instantaneous probability of having an event at time (per unit time) given that one has survived up to time.

#### 2.10.4 Kaplan–Meier Estimate

The number of observed events at t (j), j = 1, …,r. The K–M estimator of S (t) is the commonly used non‐parametric estimator of the survival function, which is designed to estimate the survival probabilities from observed survival times both censored and uncensored.

#### 2.10.5 Log‐Rank Test

The log‐rank test was used for comparing two or more survival curves in the case that the distribution is right‐skewed and censored data sets. On the other hand, the Wilcox‐on test was used when there is no censoring in the data set. The log‐rank test, developed by Mantel and Haenszel (1959), is a non‐parametric test for comparing two or more independent survival curves.

#### 2.10.6 Accelerated Failure Time (AFT) Model

Accelerated failure time (AFT) model is an alternative modeling frameworks to the PH model for the analysis of survival time data when the PH assumptions don't satisfies. AFT models measure the direct effect of the explanatory variables on the survival time instead of hazard.

In this study, AFT models were developed to model the data such as exponential, Gamma shared Frailty, Weibull, log‐logistic, and log‐normal. In each case, the univariate models were fitted for the selection of covariates statistically significant at 25% level of significance and considered in for multivariable analysis.

#### 2.10.7 Model Selection

To select the better model which appropriately fit to the given data, it was necessary to compare different models by using different techniques and methods. The AIC and BIC were the most commonly used methods for model selection criteria. Therefore, the model with the smallest value of AIC and BIC was considered as the appropriate model to fit the given data.

#### 2.10.8 Checking AFT Model Assumption

The AFT model assumption was checked using statistical tests and graphical diagnostics considering the Schoenfeld residuals. The result in current study indicates that the Schoenfeld residuals are independent of time and a graphical diagnostics indicates a random pattern against time, which is evidence for occurrence of AFT assumption. The plots of Cox‐Snell residuals can also be used in the graphical valuation of the adequacy of a fitted model. Thus, the plot of the estimated hazard rate of the Cox‐Snell residuals should give a straight line with unit slope and zero intercept if the fitted model is good. These residual are calculated as the value of cumulative risk function evaluated at observed event time $T_{i}$ [32].

#### 2.10.9 The Impact of Dropouts on Data Analysis

Patients who defaulted from treatment and further results in treatment failure and high risk of clinical events. Hence, dropout patients did not have reasons from their previous visits; therefore dropout trend was Missed Completely at Random (MCAR) [33].

### 2.11 Operational Definitions

#### 2.11.1 Early Stage of Glaucoma Disease

The early stage of glaucoma often has no symptoms. When symptoms do appear, they are typically delicate and include gradual peripheral vision loss, difficulty seeing in low light, or mild blurriness.

#### 2.11.2 Moderate Stage of Glaucoma Disease

A significant but not yet total vision loss, with noticeable peripheral vision loss, and potentially blurred vision or lightness around lights. Activities like driving or navigating crowds can become challenging at this stage.

#### 2.11.3 Advanced Stage of Glaucoma Disease

At this stage, glaucoma doesn't align with standard staging and more significant optic nerve damage, noticeable peripheral vision loss (but not yet severe tunnel vision), and potential visual symptoms like halos or difficulty in low light. At this point, more intensive treatments like laser therapy, surgery, or more aggressive medication management are often required to slow the disease and preserve vision.

#### 2.11.4 Short Duration of Treatment

Laser procedures and oral medications those can take in minutes in an office.

#### 2.11.5 Medium Duration of Treatment

Treatment focuses on lowering intraocular pressure (IOP) to preserve vision, and follow‐up visits can range from a few months to a year or more, depending on the patient's specific condition and the effectiveness of their current treatment plan.

#### 2.11.6 Long Duration of Treatment

Eye drops are a long‐term treatment that requires continuous daily use. These therapies are designed to slow the disease's progression, but it's crucial to adhere to the prescribed treatment regimen for the foreseeable future to maintain quality of life and eye vision.

##### 2.11.6.1 A Retrospective Cohort Study Design

In the current study, a retrospective cohort study was conducted where glaucoma patients (a cohort) with a shared exposure was identified. In this study, first the exposure and outcomes were established, and then analyzes historical data to determine the relationship between them [34].

##### 2.11.6.2 Statistical Tests and Levels of Significance

For the current study, the log‐rank test was used for comparing two or more survival curves in the case that the distribution is right‐skewed and censored data sets. On the other hand, the Wilcox‐on test was used when there is no censoring in the data set. The AFT model assumption was checked using statistical tests indicated here and graphical diagnostics considering the Schoenfeld residuals. The level of significance in this study was conducted using 95% CI at 2‐sided level of significance. The interpretation of AFT model was in terms of time ratio (ϕ) Data analysis for the current was conducted using SPSS and R software.

#### 2.11.7 Survival Time

This is the duration from a specific starting point (e.g., the beginning of a study) to a defined event (diagnosis of glaucoma).

#### 2.11.8 Time Ratio (phi or Φ)

In survival analysis, which is used to study the time until an event occurs (like the onset of a disease or death), a time ratio is a way to compare the survival times between two groups. A time ratio is often used in a specific type of survival model known as an accelerated failure‐time (AFT) model. This value is a multiplier that shows how a specific factor (a covariate) “accelerates” or “decelerates” the survival time. Then, a value greater than one means that the survival time is multiplied by a number greater than one, which makes the survival time longer. This indicates a protective effect against the event (glaucoma) and a lower risk. However, a value less than one means that the survival time is multiplied by a number less than one, which makes the survival time shorter. This indicates a harmful effect and a higher risk of the event (glaucoma) [35]. In contrast, another common metric in survival analysis is the Hazard Ratio (HR). The interpretation of a hazard ratio is the inverse of a time ratio. A hazard ratio of less than one indicates a lower risk and a longer survival time. A hazard ratio of greater than one indicates a higher risk and a shorter survival time [36, 37, 38].

#### 2.11.9 Ethics Approval and Consent to Participate

A statement to confirm that all methods were performed by the ethical standards as laid down in the Declaration of Helsinki. This study was approved by the Bahir Dar University Research Technical and Ethical Review Board (Ref. no Stat‐ S/166/2023). The informed consent was waived by the Bahir Dar University Research Technical and Ethical Review Board was given to Amhara region Public Health Institution (APHI).

## 3 Results

Out of the 1102 participants included in this study, about 668 (60.6%) were female and the rest were male glaucoma patients, the majority of the participants (61.7%) were rural residents and 690 (61.6%) were illiterates. Among the participants included under the current study, about 336 (30.5%) were house wife, 347 (31.5%) were farmers, 239 (21.7%) were government workers and the remaining were engaged in private business. Concerning the stage of glaucoma, 469 (42.6%) were at advanced level and 400 (36.3%) were at moderate level. Among the glaucoma patients under study, about 670 (60.8%) were also hypertensive patients and about 667 (60.5%) were diabetic patients in addition to glaucoma. In other expressions, among 670 hypertensive patients under study 526 (78.5%) became blind and 144 (21.5) became not blind. Hence, hypertension is may be one of the factors to accelerate the time to blindness to become shorter. This means, Hypertension can cause blindness by damaging the blood vessels. The reason for 56.7% of patients with no hypertension and 21.5% patients with hypertension to become blind even though patients are free from hypertension, there may be other factors lead to become blind. Finally, regarding to cup‐disk ratio about 576 (52.3%) had cup‐disk ratio of 0.7 and the remaining had cup‐disk ratio of > 0.7 (Table 2).

**Table 2:** Baseline socio‐demographic and clinical features of patient (*n* = 1102).

Variables	Categories	Survival status for blindness of glaucoma patients		Total (%)
		Blind (%)	Not‐blind (%)	
Sex	Female	458 (68.6)	210 (31.4)	668 (60.6)
	Male	150 (34.6)	284 (65.4)	434 (39.4)
Residence area	Rural	332 (48.8)	348 (51.2)	680 (61.7)
	Urban	52 (12.3)	370 (87.7)	422 (32.3)
Marital status	Living with partner	364 (55.3)	294 (44.7)	658 (59.7) 444 (41.3)
	Living without partner	141 (31.8)	303 (68.2)	
Level of education	Illiterate	463 (67.1)	227 (32.9)	690 (62.6)
	Literate	116 (28.2)	296 (71.8)	412 (37.4)
Job type	House wife	225 (67.0)	111 (33.0)	336 (30.5)
	Government work	38 (15.9)	201 (84.1)	239 (21.7)
	Private business	82 (45.6)	98 (54.4)	180 (16.3)
	Farming	233 (67.1)	114 (32.9)	347 (31.5)
Existence of Hypertension	No	187 (43.3)	245 (56.7)	432 (39.2)
	Yes	526 (78.5)	144 (21.5)	670 (60.8)
Type of treatment	Timolol	88 (32.4)	182 (67.4)	270 (24.5)
	Timolol and Pilocarpine	105 (52.5)	95 (47.5)	200 (18.1)
	Timolol and Diamox	119 (48.6)	126 (51.4)	245 (22.2)
	Timolol, Pilocarpine, and Diamox	255 (65.9)	132 (34.1)	387 (35.1)
Stage of glaucoma	Early	99 (42.5)	134 (57.5)	233 (21.1)
	Moderate	191 (47.7)	209 (52.3)	400 (36.3)
	Advanced	244 (52.0)	225 (48.0)	469 (42.6)
Duration of treatment	Short	210 (46.7)	240 (53.3)	450 (40.8)
	Medium	229 (52.2)	210 (47.8)	439 (39.8)
	Long	107 (50.2)	106 (49.8)	213 (19.3)
Existence of Diabetes	No	190 (43.7)	245 (56.3)	435 (39.5)
	Yes	302 (45.3)	365 (54.7)	667 (60.5)
Cup‐disk ratio	0.7	249 (22.6)	327 (29.7)	576 (52.3)
	> 0.7	278 (52.9)	248 (47.1)	526 (47.7)
IOP	Normal	158 (34.6)	298 (65.4)	456 (41.4)
	Not normal	325 (50.3)	321 (49.7)	646 (58.6)

### 3.1 Overall Survival Status of Glaucoma Patients

Out of the 1102 glaucoma patients followed for 60 months, about 608 (55.2%) experienced blindness, while the remaining 494 (44.8%) were censored. The minimum follow‐up period was 2.3 months, and the maximum was 60 months. The overall mean and median survival time was 30.6 and 24 months respectively.

### 3.2 Kaplan‐Meir Curve for Glaucoma Patients

**Figure 2:**
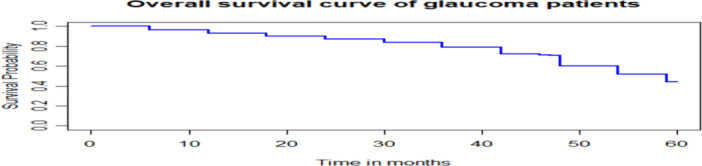
The overall estimate of Kaplan‐Meier survival curve of glaucoma patients.

The overall survivor function was declining monotonically as time in month increases (Figure 2).

### 3.3 Kaplan‐Meir Curve for Some of the Covariates

**Figure 3:**
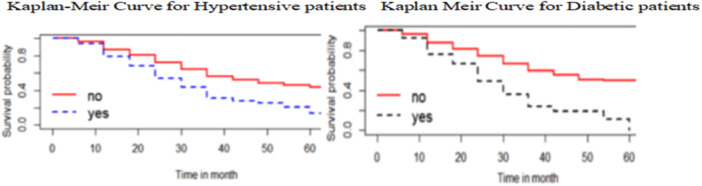
Kaplan‐Meir curve for time to blindness considering for the Hypertensive and Diabetic.

Kaplan–Meier curves for some of the covariates like hypertensive and diabetics patients under study indicated that glaucoma patients without hypertension have a significantly better survival probability (a lower risk of becoming blind) compared to those with hypertension. Similarly, glaucoma patients with diabetes have a significantly better survival probability (a lower risk of becoming blind) compared to those without diabetes (Figure 3).

### 3.4 Accelerated Failure Time (AFT) Model Fitting

As it is indicated in Table 3, the covariates sex, age in years, residence areas, level of education, Job type, existence of Hypertension, type of treatment, stage of glaucoma, duration of treatment, Cup‐disk ratio, existence of diabetes and IOP were statistically and significantly associated in the Uni‐variable AFT model analysis for the time to blindness of glaucoma patients, all the selected Uni‐variable covariates were considered as potential predictors in the multivariable AFT mode.

**Table 3:** Results of the log‐rank test for each categorical variable of glaucoma patients.

Covariates	DF	Chi‐square	*p*‐value
Sex	1	1.72	0.02*
Residence area	1	3.82	< 0.01*
Level of education	1	72.6	< 0.01*
Age	1	7.65	< 0.01*
Existence of Hypertension	1	36.7	0.01*
Job type	3	38.54	< 0.01*
Existence of Diabetic Disease	1	24.9	0.03*
Type of medication	3	86.52	0.01*
Duration of treatment	2	53.7	< 0.01*
Stages of glaucoma	2	42.63	< 0.01*
Cup‐disk ratio	1	74.5	< 0.01*
IOP	1	54.62	< 0.01*

* Stands for significant at 0.05 level of significance.

### 3.5 Test for the Robustness of Gamma Shared Frailty Model

Among the different AFT models, exponential, gamma shared frailty, Weibull, Log‐Logistic and Log normal models were compared for the current data. The AIC and BIC values for Gamma shared frailty AFT model was smallest as compared to others (Table 4). Therefore, for the glaucoma patient's data set, gamma shared Frailty AFT model was the best to fit the dataset for the current study. Hence, random component (frailty) was selected for gamma shared frailty because of its smallest AIC and BIC values. The final gamma shared frailty model is indicated in Table 5 for parameter estimates of time to blindness.

**Table 4:** Model comparison for AFT.

Model	Exponential	Gamma shared frailty	Weibull	Log‐logistic	Lognormal
AIC	2549.53	2365.45	2456.308	3523.849	3201.178
BIC	2737.342	2452.18	2615.854	4655.496	3642.844

**Table 5:** Parametric estimates for Gamma Shared Frailty AFT model.

Variables	Categories	β	S.E	*p*‐value	ϕ	95% CI for ϕ	
						Lower	Upper
Age	—	−0.3658	0.0515	0.02*	0.694	0.408	0.773
Sex of patients (Ref. = male)	Female	0.032	0.875	0.32	1.033	0.653	1.543
Education (Ref: illiterate)	Literate	0.151	0.0296	0.001*	1.163	1.097	1.232
Residence (Ref: Rural)	Urban	0.1457	0.0597	0.01*	1.157	1.049	1.274
Existence of hypertension (Ref: = Yes)	No	−0.2087	0.0958	0.01*	0.812	0.673	0.979
Existence of diabetes (Ref: = Yes)	No	−0.5023	0.0649	0.02*	0.949	0.533	0.987
Job type (Ref. = G. workers)	House wife	−0.3257	0.0549	0.02*	0.722	0.428	0.975
	Farming	−0.031	0.0058	0.01*	0.969	0.958	0.981
	Private business	−0.211	0.765	0.14	0.809	0.456	1.968
Stage of glaucoma (Ref. = Early)	Moderate	−0.234	0.0600	0.02*	0.791	0.419	0.890
	Advanced	−0.041	0.0052	0.01*	0.959	0.950	1.970
Type of treatment (Ref. = Timolol)	Timolol and Pilocarpine	0.1333	0.0644	0.06	1.143	0.907	1.296
	Timolol and Diamox	−0.1056	0.0698	0.24	0.8998	0.569	1.274
	Timolol, Pilocarpine, and Diamox	0.1422	0.0556	0.08	1.153	0.047	1.367
Duration of treatment (Ref. = Short)	Medium	0.2826	0.0801	0.03*	1.327	1.146	1.427
	Long	0.033	0.4623	0.20	1.034	0.752	1.421
Cup‐disk ratio (Ref. = 0.7	> 0.7	−0.072	0.6686	0.03	0.931	0.739	0.998
IOP (Ref. = Not‐normal)	Normal	0.056	0.0187	0.003*	1.058	1.019	1.097

*Note:* S.E represents standard error, * is statistically significance at 5% level of significance. *p* values < 0.001, indicate “*p* < 0.001”; for *p* values between 0.001 and 0.01, indicate the value to the nearest thousandth; for *p* values ≥ 0.01, indicate the value to the nearest hundredth; and for *p* values > 0.99, indicated as *p* > 0.99, β represents estimated values of each predictors (coefficient), ϕ = exp(β) indicated an acceleration factor; CI means confidence interval for ϕ, and Ref means reference.

### 3.6 Results of Multivariable Gamma Shared Frailty AFT Model

The results of the multivariable Gamma Shared Frailty AFT model (final model) is fitted as indicated in Table 5. In Table 5, it is indicated that the time ratio of greater than one (ϕ > 1) indicating a longer survival time and lower risk of glaucoma. On the other hand, the time ratio of less than one (ϕ < 1) indicating a shorter survival time and higher risk of glaucoma.

Age was a significant predictor for the time to glaucoma patients. Hence, As the age of glaucoma patients increased by 1 year, the time to blindness was accelerated by a factor of 0.7[ϕ = 0.694; 95% CI: (0.408, 0.773)] given the other covariates constant. This means that for every additional year of age, the time it takes for a patient to go blind is shortened. In this specific case, ϕ = 0.694. Since 0.694 is less than 1, it means the time to blindness is shortened or accelerated. The 95% confidence interval (CI) of (0.408, 0.773) further supports this conclusion as it is entirely below 1, confirming that this is a statistically significant finding (Table 5).

The time to blindness for literate glaucoma patients was decelerated by a factor of 1.163 compared to non‐educated patients [ϕ = 1.163, 95% CI: (1.097, 1.232)], given the other covariates constant. This means that the literate patients had a longer survival time to blindness and, therefore, a lower risk. The 95% confidence interval (1.062, 1.206) does not include the number 1, which confirms that this finding is statistically significant (Table 5).

Another significant predictor in the current study was residence area. The survival time to blindness for urban glaucoma patients was decelerated by a factor of 1.2 compared to rural patients [ϕ = 1.157; 95% CI: (1049, 1.274)]. This means urban patients had a longer survival time before going blind and a lower risk of blindness compared to rural patients The confidence interval of 95% CI: (1.049, 1.274) does not include 1, which confirms the finding is statistically significant (Table 5).

Table 5 indicated the existence of hypertension for glaucoma patients had significant contribution for the time to blindness. The time to blindness for non‐hypertensive glaucoma patients was accelerated by a factor of 0.812 compared to hypertensive patients [ϕ = 0.812; 95%: (0.673, 0.979)]. This means that non‐hypertensive patients had a a significantly better survival probability and a lower risk of the event occurring.

The other significant variable for the time to blindness response was existence of diabetes. The survival time to blindness for non‐diabetic glaucoma patients was accelerated by a factor of 0.949 compared to diabetic glaucoma patients [ϕ = 0.949; 95% CI: (0.533, 0.987)]. This means that non‐diabetic patients had a better survival probability and a lower risk of the event occurring. The 95% confidence interval (0.533, 0.987) supports this finding, as the entire range is below one, confirming a statistically significant result (Table 5).

Job type was also another significant predictor for the time to blindness. The survival time to blindness for housewives with glaucoma was accelerated by a factor of 0.772 compared to government workers (ϕ = 0.772; 95% CI: (0.428, 0.975)]. This means that housewives had a shorter time to developing blindness and a higher risk of the event occurring. The 95% confidence interval (0.428, 0.975) supports this finding. Since the entire range is less than 1 and does not include 1, the result is considered statistically significant (Table 5).

Similarly, the survival time to blindness for farmers was accelerated by a factor of 0.969 compared to government workers [ϕ = 0.969; 95% CI: (0.958, 0.981)]. This means that farmers had a shorter time to developing blindness and a higher risk of the event. The 95% confidence interval (0.958, 0.981) supports this finding. Since the entire range is less than 1 and does not include 1, the result is considered statistically significant (Table 5).

Table 5 indicated the survival time to blindness for patients in the moderate glaucoma stage was accelerated by a factor of 0.791 compared to those in the early stage [ϕ = 0.791; 95% CI: (0.419, 0.890)]. This means that patients with moderate‐stage glaucoma had a shorter time to developing blindness and a higher risk of the event. The 95% confidence interval (0.419, 0.890) supports this finding. Since the entire range is less than 1 and does not include 1, the result is considered statistically significant.

Correspondingly, the time to blindness for advanced‐stage glaucoma patients was accelerated by a factor of 0.959 compared to early‐stage patients (ϕ = 0.959; 95% CI: (0.950, 0.970)]. This means that advanced‐stage patients had a shorter time to developing blindness and, therefore, a higher risk of the event occurrence. The 95% confidence interval (0.950, 0.970) supports this finding. Since the entire range is less than 1 and does not include 1, the result is considered statistically significant (Table 5).

Cup‐disk ratio was also significant for the time to blindness in this study. The survival time to blindness for glaucoma patients with a Cup‐to‐Disc Ratio (CDR) greater than 0.7 was accelerated by a factor of 0.931 compared to those with a CDR of 0.7[ϕ = 0.931; 95% CI: (0.739, 0. 998)]. This means that patients with a higher CDR had a shorter time to developing blindness and a higher risk of the event. The 95% confidence interval (0.739, 0.998) supports this finding. Since the entire range is less than 1 and does not include 1, the result is considered statistically significant. Finally, the survival time to blindness for glaucoma patients with normal Intraocular Pressure (IOP) was decelerated by a factor of 1.058 compared to those with abnormal IOP[(ϕ = 1.058; 95% CI: (1.019, 1.097)]. This means that patients with normal IOP had a longer time to developing blindness and a lower risk of the event occurring. The 95% confidence interval (1.019, 1.097) supports this finding, as the entire range is above 1 and does not include 1, confirming a statistically significant result (Table 5).

### 3.7 Overall Goodness of Fit Test

**Figure 4:**
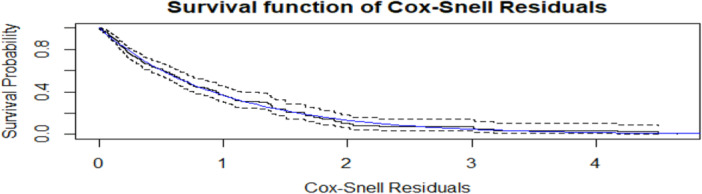
Cox‐Snell residual plots for the Gamma shared frailty AFT model for the time to blindness among glaucoma patients under treatment.

The purpose of the Cox‐Snell residual plot is to determine if the fitted model, which incorporates both the distribution assumption (Gamma shared frailty AFT model) and the covariate effects, is a good fit for the data. Black solid line (Kaplan‐Meier of Residuals) line represents the empirically estimated survival function of the calculated Cox‐Snell residuals. Blue line (unit exponential survival function) line represents the theoretical survival function of the unit exponential distribution, which is $\hat{S}\left(t\right)=e^{-t}$. On the log‐log scale (which is often implicitly used for this type of plot, or by plotting the cumulative hazard of the residuals), this is a perfect 45‐degree straight line with an intercept of zero. For a good fit, the Kaplan‐Meier curve of the residuals (black line) should closely follow the theoretical unit exponential curve (blue line). The black solid line denotes the Kaplan‐Meier estimate of the survival function of the residuals and the blue line, the survival function of the unit exponential distribution. Then, the graphical evidence from Cox‐Snell residual plot strongly suggests that the parametric assumptions (the baseline distribution) and the covariate structure of Gamma shared frailty AFT model appropriately describe the time to blindness in the glaucoma patient dataset. This result indicated that the Gamma shared frailty AFT model fitted well for glaucoma patient's dataset (Refer to Figure 4).

## 4 Discussion

The median time to blindness for glaucoma patients in this study was 24 months, with an interquartile range of 18 to 39 months. The analysis using a Gamma shared frailty Accelerated Failure Time (AFT) model showed that some variables were associated with a shorter time to blindness, while others were linked to a longer survival time.

A Gamma shared frailty AFT model revealed a significant latent or random effect (*p*‐value < 0.01). This indicates that there's heterogeneity, or unexplained variation, in the survival time to blindness among glaucoma patients across different hospitals in the region. This is likely due to differences in health care service provision. This finding is consistent with previous studies [39, 40]. This means all subjects within a group (all patients from the same hospital) share the same unobserved risk factor, or frailty. This significance confirms that the time to blindness is not uniform across all hospitals. Some hospitals likely have factors such as better quality of care, more experienced staff, or advanced equipment that lead to longer survival times for their patients, while others do not.

Age is a significant factor in the progression of glaucoma. As people age, their eye's drainage system becomes less efficient, which can lead to an increase in intraocular pressure (IOP). This increased pressure, combined with a weakened optic nerve, raises the risk of damage. The risk for glaucoma, especially open‐angle glaucoma, increases after age 40 and doubles every 10 years after age 60. Although glaucoma can affect people of all ages, it's more common and aggressive in older adults [41]. While one study confirms age as a significant predictor for the time to blindness, another study found that age had no effect on the variable of interest [42]. The potential reason for this variation might be geographic and temporal differences in study populations can influence results. Smaller sample sizes may lack the statistical power to detect a significant effect. Variations in research methods, such as data collection techniques or statistical models used, can lead to differing conclusions. This inconsistency highlights the need for further investigation to better understand the role of age in the time to blindness among glaucoma patients.

Results of the current study revealed that better‐educated glaucoma patients have a longer time to blindness. This is because education level significantly predicts the time to blindness, likely due to a better understanding of the disease and medication adherence [43]. The study revealed that patients with a better level of education have a longer time to blindness. The potential reason for this is that educated patients may have a better understanding of their condition, which leads to better adherence to prescribed medications. This finding aligns with a previous study [44], which stated that educated patients tend to have closer follow‐ups for their health compared to non‐educated patients.

In this study, residence area was a significant predictor of time to blindness. The findings show that urban glaucoma patients have a longer survival time before going blind compared to patients in rural areas [45]. The difference in survival time could be attributed to a number of factors, including environmental conditions. One potential reason is the exposure to dust particles in rural areas [46]. Higher levels of dust could contribute to eye irritation and inflammation, potentially accelerating the progression of glaucoma and leading to an earlier onset of blindness.

Farmer glaucoma patients are at risk for time to blindness because of the nature of their job which is farming exposed for different risks of their eyes. This result is also supported by the other previous study [47]. Glaucoma patients who are working in cooking foods are also exposed for smoke which is dangerous for their eyes and this indicates that the nature of job significantly affected the time to blindness for glaucoma patients in the study area [48].

Hypertension significantly affects the time to blindness in glaucoma patients. This finding is supported by a number of previous studies [49, 50], confirming that high blood pressure is a key factor in how quickly a patient with glaucoma progresses to blindness. Similarly, the presence of diabetes affects the time to blindness in glaucoma patients. This finding is consistent with other studies, indicating that diabetes is a factor in how quickly a patient with glaucoma may lose their sight [51]. Intraocular pressure (IOP) is a significant variable for the length of time it takes for a person with glaucoma to go blind [42]. A higher IOP is a major risk factor for optic nerve damage, which leads to vision loss.

### 4.1 Strength and Weakness of the Current Study

The results obtained in the current study would be helpful to identify intervention areas especially for healthcare service providers, regional policymakers towards a more effective response to the individuals with glaucoma patients under treatment. The results under the current study can be used as a bench mark for further future studies. It is also helpful for healthcare service providers in preparing health related education for awareness creation for patients while they come to hospitals for treatment.

The current study was not without limitation, this study was based on retrospective cohort study design, the data obtained from glaucoma patient's chart. However, some important socio‐demographic and clinical predictors like cataract surgery and other information were not included in the current study. Including such predictors may give additional information for the time to blindness response variable.

## 5 Conclusion and Recomendation

This study, using a Gamma shared frailty accelerated failure time (AFT) model, found that the median time to blindness for glaucoma patients in the Amhara region was 24 months. Several factors were identified as significant predictors of this outcome. Age, lack of education, and residing in a rural area were all associated with a shorter time to blindness. Conversely, patients with better IOP control, no concurrent hypertension or diabetes, and those with a lower cup‐to‐disc ratio (CDR) showed a longer time to blindness. The significant frailty effect confirmed that there is substantial variation in patient outcomes across different hospitals, likely due to differences in healthcare quality and provision.

As a recommendation, health professionals should give attention for hypertensive, diabetic and glaucoma patients with cup‐disk ratio greater than 0.7 during the follow‐up time to reduce the risk of blindness of glaucoma patients. Public Health Intervention implement targeted educational programs for glaucoma patients, especially those who are older or live in rural areas. These programs should focus on the importance of medication adherence, regular follow‐up visits, and understanding the disease. Hospitals in the region should standardize their treatment protocols for glaucoma to reduce the observed heterogeneity in patient outcomes. Training for healthcare professionals should be provided to ensure consistent and high‐quality care. Future studies should investigate the specific reasons for the variation in outcomes among hospitals. Research should focus on identifying and quantifying the impact of different healthcare practices, resources, and patient‐specific factors that were not included in this study. This will help in developing evidence‐based guidelines for improving glaucoma management in the region.

The analysis revealed a statistically significant Gamma shared frailty effect at the hospital level. This significant frailty indicates substantial unmeasured inter‐hospital variability in the time to blindness, suggesting that a patient's outcome is unduly dependent on the specific hospital providing treatment. To minimize this disparity and ensure consistent, high‐quality care, it is critically important to standardize treatment protocols across all facilities. This intervention would aim to reduce the unmeasured variation captured by the frailty term, thereby making patient prognosis more predictable based on clinical factors rather than institutional differences.

## Author Contributions

Awoke Seyoum Tegegne was participated in conceptualization, methodology, software, data curation, investigation, validation, formal analysis, supervision, visualization, writing‐original draft, and reviewing and editing. Nurye Seid Muhie was involved in conceptualization, methodology, software, data curation, investigation, validation, formal analysis, supervision, visualization, writing‐original draft, and reviewing and editing.

All authors have read and approved the final version of the article. Nurye Seid Muhie had full access to all of the data in this study and takes complete responsibility for the integrity of the data and the accuracy of the data analysis.

## Funding

The authors received no specific funding for this work.

## Ethics Statement

All methods were performed in accordance with the ethical standards as laid down in the declaration of Helsinki. Hence, an informed consent was waived by Bahir Dar University research technical and ethical review board with Ref.no Stat‐S/166/2023, as a retrospective nature of the data used in the current study. Hence, the study was approved by Bahir Dar University Research Technical and Ethical Review Board. To get the secondary data, the ethical review letter given from Bahir Dar University ethical review board was given to Amhara region Public Health Institution (APHI).

## Conflicts of Interest

The authors declare no conflicts of interest.

## Transparency Statement

The lead author Nurye Seid Muhie affirms that this manuscript is an honest, accurate, and transparent account of the study being reported; that no important aspects of the study have been omitted; and that any discrepancies from the study as planned (and, if relevant, registered) have been explained.

## Data Availability

Data available on request from the authors.
